# The Concept of Sasang Health Index and Constitution-Based Health Assessment: An Integrative Model with Computerized Four Diagnosis Methods

**DOI:** 10.1155/2013/879420

**Published:** 2013-06-12

**Authors:** Jaeuk U. Kim, Boncho Ku, Young-Min Kim, Jun-Hyeong Do, Jun-Su Jang, Eunsu Jang, Young Ju Jeon, Keun Ho Kim, Jong Yeol Kim

**Affiliations:** Medical Research Division, Korea Institute of Oriental Medicine, Daejeon 305-811, Republic of Korea

## Abstract

Sasang constitutional medicine (SCM) shares its philosophy with that of personalized medicine: it provides constitution-specific treatment and healthcare individualized for each patient. In this work, we propose the concept of the Sasang Health Index (SHI) as an attempt to assess the individualized health status in the framework of SCM. From the target population of females in their fifties and older, we recruited 298 subjects and collected their physiological data, including complexion, radial pulse, and voice, and their questionnaire responses. The health status of each subject was evaluated by two Korean medical doctors independently, and the SHI model was obtained by combining all the integrative features of the phenotype data using a regression technique. As a result, most subjects belonged to either the healthy, subhealthy, or slightly diseased group, and the intraclass correlation coefficient between the two doctors' health scoring reached 0.95. We obtained an SHI model for each constitution type with adjusted *R*-squares of 0.50, 0.56, and 0.30, for the TE, SE, and SY constitution types, respectively. In the proposed SHI model, the significant characteristics used in the health assessment consisted of constitution-specific features in accordance with the classic literature and features common to all the constitution types.

## 1. Introduction

In parallel with the prolonged life expectancy at birth in developed countries, attention in medical research has shifted from diseases with simple causes to diseases that have multiple causes and are complex and age related. Such diseases include cardiovascular diseases, metabolic diseases, nervous and psychological diseases, and cancers [1]. In this transitional era of medicine, there is a growing need for medical treatment that is more holistic and personalized rather than reductive [2].

Sasang constitutional medicine (SCM), a subdivision of traditional Korean medicine, is both holistic and patient-specific [3, 4]. The original theory of SCM was postulated by Lee, a Korean medical doctor, approximately a century ago. In SCM, every person can be categorized into one of the four Sasang constitution (SC) types according to his/her inherited characteristics, such as his/her temperament profile, physiological and pathological features, physical characteristics, and response to drugs and treatment. The four SC types are *Tae-Eum* (TE, *Greater Yin*), *So-Eum* (SE, *Lesser Yin*), *So-Yang* (SY, *Lesser Yang*), and *Tae-Yang* (TY, *Greater Yang*). An individual's constitution type is determined by the functional balance between the production and consumption of Qi and body fluid or between food intake and waste discharge. The TE (TY) type, which is also called the liver (lung) type, is hyperactive in the production and storage (consumption) of Qi and body fluid and hypoactive in the consumption (production and storage) of Qi and body fluid. On the other hand, the SE (SY) type, which is also called the kidney (pancreas) type, is hyperactive in waste discharge (food intake) and hypoactive in food intake (waste discharge) [5–8].

The basic goal of SCM treatment is to recover the balance between hypoactive organs and hyperactive organs which are predetermined by one's constitution. Therefore, in SCM, the first and most important step of medical treatment is correctly diagnosing the patient's constitution. In clinics, complicated sociocultural interactions mean that the indicators of the four categorical factors, ranging from temperament profile to the susceptibility to herbal treatments, may show competing features of more than one SC type, thereby hampering the correct diagnosis of the patient's constitution and diminishing the effectiveness of SCM. 

To attain more reliable diagnoses of patients' constitutions, many attempts have been made to standardize the diagnostic process and make it more objective. Through decades of effort, facial metrics [9–13], body shape analysis [14–17], vocal features [18–20], and questionnaires [21–23] have been shown to be effective in the classification of SCs. These efforts resulted in prototypical tools for SC classification, such as the Phonetic System for Sasang Constitution 2002 [24, 25] and the Questionnaire for Sasang Constitutional Classification II (QSCCII) [23]. 

These early prototypical tools for SC classification were not sufficient for practical use, because of limited validity of the imbedded classification models resulting from the analysis of a single component, such as voice or questionnaire responses, and the small number of narrowly focused clinical trials used to develop the models. To overcome these limits, an integrative SC analysis tool (SCAT ver. 1.0) was developed recently [26]. To diagnose Sasang constitutions, the SCAT system followed the logic of medical doctors' decision-making based on the combination of facial features [27], body shape [28], vocal features [29], and questionnaire responses [23]. To make the SCAT system generalizable, the SC decision-making model was developed based on the constitutional data of 2,973 male and female subjects older than 15 years, which was collected between 2007 and 2011 at 23 clinical sites around Republic of Korea.

Research activities related to improving the objectiveness of the SCM have primarily focused on the SC classification. With a reliable model for classifying the Sasang constitutions, the assessment of the health status specific to each constitution may be a natural product of SCM research and development. Very recently, a few studies have attempted to create the objective assessment of constitution-dependent health status. Clinical studies of approximately 300 female subjects have shown that some aspects of facial color [30], skin properties [31], and pulse properties [32] differed significantly between healthy subjects and unhealthy subjects in a constitution-specific manner.

In this work, we developed the Sasang Health Index (SHI), a decision-making model that was constitution specific and that estimated the health condition level of each constitution independently. Because of limited resources, we focused on female subjects in their fifties and above to attain the validity of the SHI decision model within the selected population range. To make the SHI model integrative, we combined five core components of the four methods of diagnosis: facial features; vocal features; radial pulse properties; skin properties measured at the arm and the back of the hand; and a questionnaire developed for Sasang health estimation. The SHI model was developed using a two-stage process; first, we made individual SHI (SHI_ comp _) models for every diagnostic component, and then we used the SHI_ comp_ scores as the parameters for the integrative SHI (SHI_ sum _) model based on the regression of the least absolute shrinkage and selection operator (LASSO).

## 2. Health Levels in SCM and the Concept of Sasang Health Index

Lee proposed in his theory of SCM that human health could be categorized into 8 health levels (*Myong-Maek-Sil-Soo (Ming-Mai-Shi-Shu)*, Life Quality Index). Every health level was further divided into 3 substages. Detailed descriptions of the 8 health levels and the 24 more detailed health stages can be found in *Dongeuisusebowon-Sasang-Chobonkwon (Dongyishoushibaoyuan-Sasang-Caobenjuan)*; a summary is presented in Table 1 [4]. The upper four health levels ranging from *Kangnyeong* to *Shinseon *are states of no illness, and the lower four levels ranging from *Woekam *to *Weekyong* can be evaluated in terms of the duration and severity of illness over a year long period.

According to Lee's theory, a patient at the *Woekam* level does not need any medication (daily self-care is sufficient); as his/her illness progresses to *Naesang* or *Noeok*, he/she becomes increasingly dependent on medication. At the worst health level, *Weekyong*, no medication will be effective in improving the illness.

Health screening programs for the general population are a worldwide trend. According to the 2010 report for Korea's Nationwide Health Screening Program (NHSP), the proportions of healthy, subhealthy, and diseased people were 8.1%, 61.8%, and 30.1%, respectively [33]. Similarly, according to a recent survey, approximately 75% of the citizens of Beijing and Shanghai were reported to be in a subhealthy state [34]. The subhealthy (normal B) state was defined as a state at which no medication or minimal level of medication is needed, but preventive self-care (e.g., by improving eating habits or lifestyle) is recommended.

Let us interpret the SCM health levels in connection with the health status categories used in the NHSP. The *Woekam* level of SCM may be mapped onto the subhealthy state in the NHSP because, for both levels, daily self-care without medication can improve or maintain the individual's health condition. By interpolation, we continue to interpret the *Kangnyeong* level as the healthy state, the *Naesang* level as the slightly diseased state, and the *Noeok* level and below as heavily diseased states. In modern society, therefore, the majority of the population can be classified into the *Kangnyong, Woekam, *or* Naesang* levels [33].

A fascinating feature of the SCM is that both the major phenotypical symptoms of health-level degradation and the treatment methods are constitution specific, as summarized for some features in Table 2. For instance, sweating during light daily activity is a sign of good health for a TE person, but it is a sign of bad health for an SE person. Similarly, when health condition becomes bad, an SE person is likely to have indigestion and a hazy yellow complexion, while an SY person is likely to have constipation and a pale or dark complexion.

To date, the constitution-specific features used to determine an individual's health status have arisen from the classical literature and clinical experiences and have rarely been studied for verification or objectiveness. In this work, in an attempt to objectify and provide scientific evidence for SCM's phenotypical health theory, we suggest the concept of a Sasang Health Index (SHI): high SHI scoring for good health and low SHI scoring for bad health. Because the majority of the population belongs to either a healthy (*Kangnyong*), subhealthy (*Woekam*), or slightly diseased (*Naesang*) health category, we aimed to develop a constitution-specific SHI model that could provide a guideline for the *Kangnyong* to *Naesang* levels, which are equivalent to the healthy, subhealthy, or slightly diseased NHSP categories, respectively.

In accordance with the diagnostic theory of SCM, the SHI health estimation model was based on the collective features of four methods of diagnosis. We used electronic devices to measure and extract face, voice, pulse and skin features, and questionnaire responses. In the following section, we outline the clinical study, measurement devices, and variables used in the SHI model.

## 3. Methods

### 3.1. Study Design

**Figure 1:**
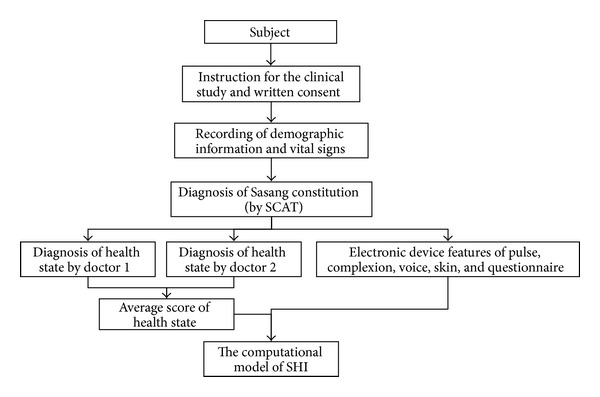
Outline of the clinical study to obtain the SHI model.

The procedure of the clinical study is outlined in Figure 1. Informed written consent was obtained from the volunteers prior to their entry into the study. Information about the subjects' Sasang constitution was acquired using the Sasang Constitution Analysis Tool (SCAT) developed by Korea Institute of Oriental Medicine. The SCAT is an automated SC decision-making system based on an integrative combination of the subject's facial, body shape, vocal, and questionnaire response features [26]. Two Korean medical doctors with at least five years of clinical experience independently diagnosed the subjects' health states using the visual analogue scale (VAS). We determined the constitutional health score by averaging the VAS scores assigned by the two doctors. Finally, by analyzing the correlation between the constitutional health score and the integrative features of the five decision-making components (complexion, pulse, voice, skin, and questionnaire response), we developed the Sasang Health Index (SHI). More details about the subjects, the procedure used to develop the SHI model, and the characteristic features of the five decision-making components are described below. The clinical study was conducted for 3 months in 2012 and was approved by the institutional review board at the Oriental Hospital of Daejeon University, Korea (IRB no. M2012-01).

### 3.2. Study Subjects

The study was conducted on female volunteers aged 50 to 75 years with no mental disorders or deformities of the measurement positions of the face, voice, skin, and radial artery. Those who had ingested alcohol within six hours or smoked within one hour of the examination were excluded. Prior to the clinical study, demographic information and such vital signs as height, weight, blood pressure, pulse rate, and temperature were recorded.

### 3.3. Health Assessment

Two Korean medical doctors with at least five years of clinical experience participated to diagnose the subjects' health states. To determine a subject's health state, doctors used the four methods of diagnosis (observation, auscultation and olfaction, inquiry, and pulse feeling and palpation). For the health evaluation, doctors used a VAS scoring with scores ranging from 0 (seriously ill) to 100 (complete physical and mental well-being). For the calibration of the participating doctors' VAS scores, two reference scores of 80 and 40 were used to separate healthy subjects from unhealthy subjects and subhealthy from diseased subjects. The healthy subjects were scored 80 or higher, the subhealthy subjects were scored between 40 and 80, and the diseased subjects were scored less than 40. Subjects who were diagnosed with light common aged-related symptoms such as borderline hypertension which could be controlled by daily preventive self-care (and minimal level of medication) were categorized into the subhealthy group, and more severe symptoms which required some medication were categorized into the diseased group. The health state assessments by the two doctors were done blindly to each other. The constitutional health score was then determined by averaging the VAS scores assigned by the two doctors.

### 3.4. SHI Model and Statistical Method

The average VAS scores assigned by two Korean medical doctors were used as the standard health scores for each constitution. To obtain the computational model of SHI that best fits the doctor-assigned standard health scores, we applied a linear regression using the least absolute shrinkage and selection operator (LASSO) method. The LASSO method is beneficial for developing a stable regression model with few features and a low risk of overfitting and multicollinearity [35]. The SHI model was developed using a two-stage process. First, we obtained SHI regression models for the five independent diagnostic components (complexion, pulse, voice, skin, and questionnaire responses) using the following formula:

(1) $$\begin{array}{c} {\text{SHI}}_{\text{ comp }}=\alpha_{i,0}+\sum_{i=1}^{N}\alpha_{i,j}\times x_{i,j}, \end{array}$$

where SHI_ comp_ is the health scores for the *i*th diagnostic component and *x*_*i*,*j*_ is the *j*th feature variable of the *i*th device. The terms *α*_*i*,0_ and *α*_*i*,*j*_ are the error terms and the regression coefficients.

By repeatedly applying the LASSO method, we obtained the final SHI model, where the individual SHI_ comp_ score obtained with (1) was used as a contributing variable. Age and BMI were also included. The integrated model of SHI takes the form of

(2) $$\begin{array}{c} {\text{SHI}}_{\text{sum}}=\beta_{0}+\;\sum_{i=1}^{5}\beta_{i}{\;\times \;\text{SHI}}_{\text{ comp }}+\beta_{6}\times \text{age}+\beta_{i}\times \text{BMI}, \end{array}$$

where SHI_sum_ is the final health score for the integrated diagnostic components.

SPSS 14.0 (SPSS Inc., USA) and the *R*-statistical analysis tool were utilized to analyze the significance levels of each device variable and to develop regression models using LASSO. The accuracy of the model was tested using a 10-fold cross-validation method.

Before we applied the LASSO method, we preprocessed to select candidate variables with a significance level below *P* = 0.2 (determined with Student's *t*-test) to be used in the SHI model. To analyze the questionnaire responses, we used factor analysis. Factor analysis efficiently identifies groups of highly correlated variables and their relationship to the target variable. Factor analysis describes the covariance relationship among many variables in terms of a few underlying latent quantities [36].

### 3.5. Variables of Each Device

#### 3.5.1. Complexion Variables

**Figure 2:**
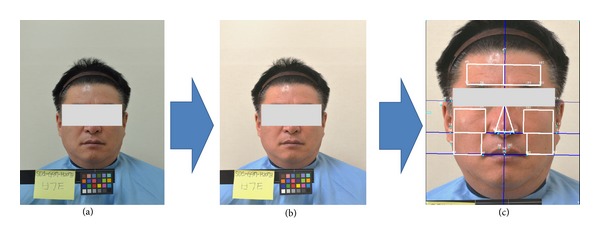
Image processing of a facial image: (a) original photographic image, (b) color-corrected image based on a standard color chart, and (c) the final facial image to analyze the complexion.

We developed an automated program to extract facial features using standard image processing techniques [30]. To obtain facial images that are robust against lighting conditions, we employed a color correction technique that used a standard color chart [30]. As depicted in Figure 2, we divided the facial domain into 10 sectors; left (FhL), right (FhR), and overall (FhW) forehead, left-up (ChLU), left-down (ChLD), left-overall (ChLW), right-up (ChRU), right-down (ChRD), and right-overall (ChRW) cheek, and nose (Nose).

At each sector, the averages (avg) and standard deviations (std) of the three components of brightness (Y), red (Cr), and blue (Cb) were calculated. We used the YCrCb color representation to overcome RGB representation's high sensitivity to lighting conditions. In total, 60 complexion variables were extracted from the FhL_Y_avg (the average of Y-component at the left forehead area) to the Nose_Cb_std (the standard deviation of the Cb-component at the Nose area), as shown in Table 3 [30].

#### 3.5.2. Radial Pulse Variables

**Figure 3:**
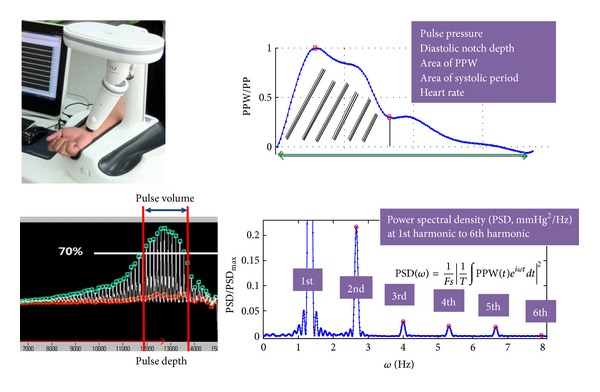
Radial pulse measurement from KIOM-PAS device and feature variables.

The raw radial pulse signal was acquired using the KIOM Pulse Analysis System (KIOM-PAS v2.0) developed at the Korea Institute of Oriental Medicine [37]. As Figure 3 shows, 21 pulse variables were extracted at the *Gwan (Guan)* locations of the left and right arms. These variables included 8 time series features, 4 tonometry-based features, and 10 features of the power spectral density (PSD).

The time series features of the radial pulse were heart rate (HR), pulse pressure (PP), time and amplitude of diastolic notch (t4, h4), t4 at heart rate 75 (t4_HR75), and 3 areal variables in waveform-time domain at HR75, that is, the area of systolic period (Asys_HR75), the area of diastolic period (Adias_HR75), and the area of one heartbeat (Aall_HR75). The tonometry-based features were pulse depth index (PDI), pulse volume index (PVI), and average pulse amplitudes within the PVI domain (PPW_PVI). The Fourier space variables were the PSD at the fundamental frequency (PSD_w1), the PSDs at harmonic frequencies relative to the fundamental frequency (PSD_w2_w1 to PSD_w7_w1), the PSD integral up to 10 Hz (PSD_0_10 Hz), and ratio of high-frequency PSD to low-frequency PSD (PSD_10_50 Hz). The pulse variables were labeled “L_variable” for a left Gwan variable or “R_variable” for a right Gwan variable [32].

#### 3.5.3. Vocal Variables

To analyze vocal features, we recorded five vowels “a,” “e,” “i,” “o,” and “u” and a sentence (repeated twice) using a wave file (.wav) with a 16-bit integer monosetting and a 44.1 kHz sampling frequency. The recording environment and procedure were strictly controlled by a standard operating procedure [38]. From the acquired data, we determined a set of candidate vocal features, including fundamental frequency (F0), variations of F0 (jitter) and amplitude (shimmer), relative average perturbation and pitch perturbation quotient as measures of frequency variation, frequencies and bandwidths of low-order formants, and duration of sentence utterance (see Table 4).

#### 3.5.4. Skin Variables

Skin physiological characteristics such as viscoelasticity and roughness were used to analyze health states. To observe the viscoelastic properties of the skin, the elasticity coefficient (*E*), elastic hysteresis (*E*_hys_), and viscoelasticity coefficient (*V*_*E*_) were calculated. Dermaflex (CORTEX TECHNOLOGY, Denmark), which has high sensing repeatability [39], was used to measure *V*_*E*_ and *E* at the center position of the inner side of the arm. The *E*_hys_ was defined as the difference between the first elasticity coefficient value and the third one. To estimate the roughness of the skin's surface, the area of the wrinkle on the back of the left hand (Wrinkle_hand) was computed from a zoomed-in, 10 times magnified image.

#### 3.5.5. Questionnaire Variables

A questionnaire for evaluating the Sasang health level was recently developed based on the SCM classic literature [3]. The questionnaire consisted of 87 questions examining health conditions and the physical and psychosocial aspects of health-related activities. Specifically, 52 questions addressed physical conditions/functions and physical activities, 33 questions addressed psychosocial conditions and activities, and 2 questions were about self-estimates of the subject's health level [40].

Because the original questionnaire was designed with huge items to explore adequate measures for health state according to SC types, some items were invalidated, unreliable, or redundant. We employed exploratory factor analysis (EFA) with combination of principal component analysis and promax rotation to extract conceptually well-defined latent factors. If some of grouped items within a factor showed (a) inexplicable, (b) invalidated, or (c) unreliable, those items were excluded. Based on the result of EFA, all items were reorganized in accordance with the conceptual factors. Cronbach's alphas were provided to test the reliability of each item.

From the result of EFA, as summarized in Table 5, we found 11 interpretable physical factors (7 factors related to physical conditions and 4 related to physical activities) that accounted for 51.4% of the variance in physical conditions and activities and 5 psychosocial factors (4 psychological and 1 social conditions) that accounted for 50.6% of the variance in psychosocial conditions/activities. The 11 physical factors consisted of physical discomfort (9 questions, Cronbach's alpha = 0.772); exercise capability (2 questions, Cronbach's alpha = 0.752); defecation (3 questions, Cronbach's alpha = 0.734); perspiration (3 questions, Cronbach's alpha = 0.743); digestion (2 questions, Cronbach's alpha = 0.600); discomfort after eating, defecation, and urination (3 questions, Cronbach's alpha = 0.509); sleep habits (6 questions, Cronbach's alpha = 0.779); exercise habits (2 questions, Cronbach's alpha = 0.799); body warming habits (2 questions, Cronbach's alpha = 0.673); eating habits (2 questions, Cronbach's alpha = 0.727); and unhealthy period (1 question). The 5 psychosocial factors consisted of sociability (14 questions, Cronbach's alpha = 0.846), emotional stability (7 questions, Cronbach's alpha = 0.819), extroversion and broad-mindedness (3 questions, Cronbach's alpha = 0.691), introspection and preparation (4 questions, Cronbach's alpha = 0.772), and addiction (2 questions, Cronbach's alpha = 0.843). The details of the question items will be discussed elsewhere [40].

## 4. Results and Discussion

### 4.1. Population Analysis

We analyzed 298 female subjects aged 50 years or older. Using SCAT to determine the subjects' constitutions, we found that 100 subjects were TE types, 72 subjects were SE types, and 126 subjects were SY types. The TY type subjects were considered outliers; the estimated proportion of TY types in the general population is approximately 0.01% [3]. The subjects were 56.3 ± 4.9 years old on average (±SD). Their average height was 156.1 ± 4.9 cm, and their average weight was 58.4 ± 8.1 kg, resulting in an average BMI of 23.9 ± 3.0 kg/m^2^.

The subjects' demographic information is shown in Table 6. The heart rate and body temperature were within the normal range and showed no significant differences among constitutions. In contrast, there were significant differences between SC types in BMI and systolic and diastolic blood pressures (one-way ANOVA test). In particular, the TE type subjects were distinguished by high BMIs and high systolic/diastolic blood pressures compared to the SE and SY type subjects. At advanced ages, subjects with high BMIs have a high risk of the metabolic syndromes including hypertension, hyperlipidemia, and diabetes, which accompany high systolic/diastolic blood pressures. Therefore, the high BMI and high systolic/diastolic blood pressures of the TE subjects were an expected consequence resulting from the TE type's intrinsic characteristic of hyperactive storage of Qi and body fluid and hypoactive consumption of Qi and body fluid [5].

### 4.2. Health-Level Distribution among SC Groups

Two participating doctors used four diagnostic methods and evaluated the subjects' health states independently of each other. For the health evaluation, we used a VAS scoring system with scores ranging from 0 to 100 (see Section 3.3). The intraclass correlation coefficient (ICC) between the two doctors' independent diagnostic VAS scores was 0.95, indicating that the two doctors used consistent references to evaluate the health state.

Table 7 shows the population distribution of healthy and unhealthy (subhealthy and diseased) subjects and the average of the two doctors' VAS scores for each Sasang constitution. One-way ANOVA test showed that the VAS score was significantly different among the three SC groups: VAS (TE) < VAS (SE) < VAS (SY). Similarly, the proportion of healthy subjects was lowest in the TE group and highest in the SY group, while the proportion of diseased subjects was highest in the TE group and lowest in the SY group. Approximately 55% of the unhealthy subjects were diagnosed with an indicator of metabolic syndrome, such as hypertension, hyperlipidemia, and diabetes. Hypothyroidism, digestive dysfunction, degenerative arthritis, and osteoporosis were the other major reasons for health degradation. As mentioned previously, high BMI of the TE group is a major cause of metabolic syndrome and arthritis, which provides a reasonable explanation for the high proportion of unhealthy subjects and the low health scores of the TE group compared with other SC groups.

### 4.3. Classification Model for Sasang Health Index (SHI)

#### 4.3.1. The SHI Model

To obtain a model of Sasang Health Index (SHI), we used the LASSO regression method (see (1) and (2)). First, we applied (1) to create regression models (SHI_ comp _) for individual diagnostic components. The variables were normalized by the *z*-transformation. The resulting LASSO estimators with corresponding significant variables for each diagnostic component are shown in Table 8.

We continued to apply (2) to obtain a model of the integrative Sasang Health Index (SHI_sum_). Because BMI and age significantly affect the health state of each SC group, these two factors were considered in the model, too. Each SHI_ comp_ score was normalized using the *z*-transformation. The result of a 10-fold cross-validation test is shown in Table 9. The adjusted *R*-square, which is the coefficient of determination modified to adjust for the number of explanatory terms in the model, shows that the SHI regression model has moderate accuracy for the TE (adj. *R*-square = 0.51) and SE groups (adj. *R*-square = 0.56) and lower accuracy for the SY group (adj. *R*-square = 0.30).

#### 4.3.2. Interpretation of the SHI Model for Each SC Type

The equal orders of magnitude of *β*-components presented in Table 9 indicate that each diagnostic component contributed almost equally to SHI_sum_. Because the variables in Table 8 and the SHI_ comp_ scores in Table 9 were normalized using the standard *z*-transformation, the significance of each variable in Table 8 is proportional to the magnitude of the associated *α*. In the following, we interpreted highly contributing variables with large coefficients *α* in Table 8 to understand the result of the SHI model.

To help understand the resulting SHI model, in Table 10, we listed the major LASSO estimators with a short description of the estimators' change related to health degradation in each SC group. For all the SC types, changes in color components became more severe with degraded health conditions. According to the classic literature, patients' facial color and glossiness change depending on symptoms or their appearing locations. For instance, with degraded health conditions, the complexion becomes less glossy, and the accompanying changes in color convey the following health information: the red shift indicates cardiac dysfunction, hazy yellow indicates digestive organ dysfunction, pale color indicates lung or bronchial disease, dark color indicates kidney disease, and the blue shift indicates liver problems [41]. In our clinical study, however, we did not observe significant changes of colors between healthy and unhealthy groups except in the case of the TE group, in which unhealthy members exhibited dark color in the nose. Instead, we observed significant increases in the variance of colors and brightness.

To date, there has been no technical report that could distinguish glossy complexion from pale complexion. Regarding color variance, a wrinkled or freckled person will be subjected to a higher standard deviation compared with a wrinkle-free or freckle-free person for a given average color intensity. For these reasons, we encountered difficulty in providing a detailed interpretation for the variance of colors and brightness in the complexion.

In terms of the radial pulse characteristics associated with a degraded health condition, for the TE group, the radial pulse became more deep-lying and forceful, the pulsatile volume during the systolic period was increased compared with the diastolic period, and the PSD at a high harmonic frequency was reduced. For the SE group, the pulse became more deep-lying. All of these are features related to the increased cardiac load required to compensate for cardiovascular system dysfunction. Approximately 55% of the unhealthy subjects in our study group were diagnosed with hypertension, hyperlipidemia, or diabetes, which implied that the indicators of metabolic syndrome determined the unhealthy subjects' overall tendencies. Moreover, this observational study showed that the TE group was characterized by a higher ratio of unhealthy versus healthy members compared with the other SC groups, most likely because of the high BMI that characterizes the TE group. Pulse is the most sensitive indicator of cardiovascular function among the diagnostic components. Because of the high proportion of metabolic syndrome in the TE group compared to other SC groups, therefore, the significant features of pulse diagnosis were thought to be observed prominently in the TE group with a tendency toward increased cardiac load. We note that the reduction of PSD at high harmonic frequencies was also observed in a recent clinical study with palpitation patients [42].

In the aspect of skin properties, wrinkle density was decreased for the unhealthy TE subjects, and the skin elasticity was decreased for the unhealthy SY subjects. By a feasibility test, we found that the reduced wrinkle density was related to the disappearance of fine wrinkles between furrows [31]. When a fatty skin becomes thinner, it will be featured by reduced elasticity and resilience, and fine and shallow wrinkles are likely to be more difficult to observe. Therefore, the decreased wrinkle density for the unhealthy TE group and decreased elasticity for the unhealthy SY group are both in compliance with the thinned skin feature, which is in agreement with Table 2 and the literature [4].

The vocal features showed increases in the frequency variation, bandwidths of the 1st and 2nd formants, shift of the fundamental frequency at the 10th percentile (sF10) into a lower frequency for unhealthy TE subjects, and increased ratio of the frequency intervals between a high to the median and the median to a low fundamental frequency (sFHL) for unhealthy SY subjects. These results imply that, with degraded health status, a TE subject would speak with more vibrant and low-pitched tone, and an SY subject would speak more variation in high-frequency regime compared with low-frequency regime, which agreed with clinical experiences.

With degraded health status, the questionnaire response showed that (1) the TE type subjects were featured by decreased exercise capability, worsened sleep habits, and decreased tendency of extroversion and broad-mindedness; (2) the SE type subjects were featured by low self-estimation of health state, low exercise capability, indigestion, worsened sleep habits, and increased unhealthy period; and (3) the SY type subjects were characterized by low self-estimation of health state, improved eating habits, and increased tendency of extroversion and broad-mindedness.

We note that indigestion was a distinctive feature of heath degradation for the SE type subjects in accordance with the literature (see Table 2). Extroversion (cowardice) is a natural temperament of SY (TE) type persons [5], and we found that the extrovert (cowardly) tendency became stronger once a SY (TE) type person was out of shape. Here, we interpreted decreased extroversion as increased cowardice. An enhanced tendency of natural temperament with degraded health condition is in agreement with the clinical experience in SCM. Similarly, an unhealthy SY type person would have improved eating habits, while the opposite would occur for a TE type person. This seemingly contradictory result could be understood as follows. Because an SY type person naturally has good digestive function [5], good eating habits are not a necessity while he/she is in good shape, but improved eating habits become necessary once he/she is out of shape.

## 5. Conclusions

The advance of genomics and biotechnology has opened the possibility of patient-specific medical treatments that offer high efficacy and the least-possible adverse effects; this field of medical science is called personalized or individualized medicine [1, 42]. Based on an individual's symptom identification in molecular level, the personalized medicine will allow physicians to make the right patient-care decisions and allow patients the opportunity to make informed and directed lifestyle decisions for their future well-being [43]. Sasang constitutional medicine (SCM) is a prototype of personalized medicine, as it offers constitution-specific diagnoses and treatments. In SCM, doctors use several phenotypical aspects to determine a patient's constitution and constitution-specific symptoms. Therefore, the health level of each patient will be constitution specific.

In this work, we proposed an efficient method for assessing the health level of individuals belonging to each SC type. The target population was females in their fifties to seventies in Republic of Korea who were healthy, subhealthy (no medication is needed), and slightly diseased (only a supportive level of medication is needed). We obtained very high concordance for the health estimations made by the two doctors (ICC = 0.95). To obtain an integrative model for the health-level estimation, we combined five diagnostic components: complexion, pulse, skin, voice, and questionnaire response features. Using an LASSO regression method, we developed the Sasang Health Index (SHI), which showed that the health level of each Sasang constitution type could be estimated with the adjusted *R*-squared of 0.56 for the SE type, 0.50 for the TE type, and 0.30 for the SY type.

In the proposed SHI model, indicators of health degradation were found to differ among the SC types. In the TE type, degraded health was accompanied with pulse characteristics indicative of increased cardiac load, a thinned skin feature, vocal features indicating more vibrant and low-pitch tone, and questionnaire responses showing decreased exercise capability, worsened sleep habits, and decreased tendency of extroversion and broad-mindedness. In the SE type, increased pulse depth and questionnaire responses showing low exercise capability, indigestion, and worsened sleep habits were found features for health degradation. On the other hand, in the SY type, a thinned skin feature, more variant vocal feature in high-frequency regime, and questionnaire responses showing improved eating habits and increased tendency of extroversion and broad-mindedness were indications of degraded health. Some of our findings were in accordance with the classic literature, but some other constitution-specific health indicators must still be verified and explained with more extensive and focused studies.

Our work is an innovative attempt to evaluate the constitution-specific health status using five diagnostic components (complexion, pulse, voice, skin, and questionnaire responses) based on the SCM theory. The study subjects were elderly females in their 50s to 70s with not-so-severe health problems such as hypertension, diabetes, and hyperlipidemia. We believe that the concept of the SHI and constitution-based health assessment model can be broadly applied beyond the study population. However, the specific results discussed here may not be generalized to other gender and age groups and more severe disease groups. Studies on more constitution-specific diseased subjects across broader age and gender spectra will provide practical results with extended coverage. Our findings will contribute to making SCM more objective and may eventually advance the realization of personalized medicine.

## Figures and Tables

**Table 1:** The 8 health levels and 24 health stages according to *Dongeuisusebowon-Sasang-Chobonkwon* [4].

Health level (Life Quality Index)	Stage	Symptom (over a one-year period)	Medication and healthcare recommendations
*Shinseon (Shenxian) *	I, II, and III	No illness at all	—
*Chongrang (Qinglang) *	I, II, and III		
*Qoekyong (Kuaiqing) *	I, II,and III		
*Kangnyeong (Kangning) *	I, II, and III		

*Woekam (Waigan) *	I II III	Duration of illness < 3 months	No medication is needed (daily self-care is enough)

*Naesang (Neishang) *	I II III	Duration of illness < 6 months	Primarily daily self-care, with supportively medication

*Noeok (Laoyu) *	I II III	Duration of illness < 9 months	Primarily medication, with supportive daily self-care

*Weekyong (Weiqing) *	I	One month illness-free with medication and good healthcare	No medication can improve the illness
	II	Incurable state of disease; medication temporarily alleviates the illness	
	III	No medication can alleviate the illness	

**Table 2:** Representative phenotypical signs of health states for each of the SC types described in *Dongeuisusebowon-Sasang-Chobonkwon* [4].

	TE	SE	SY	TY
Healthy sign General healthy sign	Perspiration	Good digestion	Good bowel movements	Smooth urination
Facial feature	Glossy purple complexion	Glossy purple complexion	Serene blue complexion	Serene fair complexion
Pulse feature	Long and tight	Smooth and weak	Floating and rapid	—
Vocal feature	Loud and raspy Gravelly and throaty	Toneless and soft spoken Lilting and mellow	High pitched and hasty Merry and clear	Talkative and hasty High pitched, clear, persuasive, loud, and resonant
Skin feature	Hazy and fat	Glossy and thin	Hazy and fat	Glossy and thin
Sleep habits	Loud and deep breathing, powerful tossing and turning	Loud and deep breathing, powerful tossing and turning	Smooth breathing, restful, calm, and heavy posture	Smooth breathing, decent, calm, and heavy posture
Sweating	Profuse sweating	No/little sweating	No/little sweating	Profuse sweating

Unhealthy sign General unhealthy sign	Absence of perspiration	Indigestion	Constipation	Musculoskeletal weakness Emesis
Facial feature	—	Hazy yellow complexion	Pale or dark complexion	Dark complexion
Skin feature	Glossy and thin	Hazy and fat	Glossy and thin	Hazy and fat
Sweating	No/little sweating	Profuse sweating	Profuse sweating	No/little sweating

**Table 3:** Facial variables in the YCrCb color space.

Facial variable	Label	Field	Description
Sector_color_stat	Sector	[FhL, FhR, FhW, ChLU, ChLD, ChLW, ChRU, ChRD, ChRW, and Nose]	Fh: forehead, Ch: cheek, Nose: nose, L: left, R: right, W: overall, LU: left-up, LD: left-down, LW: left-overall, and RW: right-overall
	Color	[Y, Cr, Cb]	Y: brightness, Cr: red color, and Cb: blue color
	Stat	[avg, std]	avg: average, std: standard deviation

**Table 4:** Vocal variables with *x* *∈* [a, e, i, o, u].

Vocal variable	Description
xF0	Average fundamental frequency
xJITT	Jitter (cycle-to-cycle variation of fundamental frequency)
xRAP, xPPQ	Relative average perturbation and pitch perturbation quotient (frequency variation characteristics)
xSHIM	Shimmer (cycle-to-cycle variation of amplitude)
xAPQ	Amplitude perturbation quotient
xF1, xF2, and xF3	1st, 2nd, and 3rd formant frequencies
xBW1, xBW2	Bandwidths of the 1st and 2nd formants
sDT	Duration of sentence utterance
sF10, sF50, and sF90	Fundamental frequencies at the 10th, 50th, and 90th percentiles (sentence)
sFHL	Ratio of the frequency intervals between a high to the median and the median to a low fundamental frequency = (sF90 − sF50)/ (sF50 − sF10)

**Table 5:** Factor analysis and selected factors that accounted for more than 50% of the variance.

Category	Factor (number of participating questions)
Physical	Physical discomfort (9)
	Exercise capability (2)
	Defecation (3)
	Perspiration (3)
	Digestion (2)
	Discomfort after eating, defecation, and urination (3)
	Sleep habits (6)
	Exercise habits (2)
	Body warming habits (2)
	Eating habits (2)
	Unhealthy period (1)

Sociopsychological	Sociability (14)
	Emotional instability (7)
	Extroversion and broad-mindedness (3)
	Introspection and preparation (4)
	Addiction (2)

**Table 6:** Demographic data grouped by Sasang constitution type.

Physiological data	Sasang constitution type		
	TE	SE	SY
Number (*n*)	100	72	126
Age (yr)	57.4 ± 5.5	56.7 ± 4.9	55.2 ± 4.4
Height (cm)**	157.1 ± 5.0	156.3 ± 4.7	155.3 ± 4.8
Weight (kg)**	66.2 ± 7.0	52.4 ± 5.5	55.8 ± 5.1
BMI (kg/m^2^)**	26.8 ± 2.6	21.4 ± 2.0	23.2 ± 1.8
Systolic blood pressure (mmHg)**	127.8 ± 17.4	115.1 ± 14.2	118.4 ± 15.8
Diastolic blood pressure (mmHg)**	76.7 ± 10.2	72.1 ± 9.1	72.7 ± 10.2
Heart rate (beats/min)	69.3 ± 8.4	69.9 ± 7.7	68.7 ± 8.3
Body temperature (°C)	36.4 ± 0.3	36.4 ± 0.3	36.4 ± 0.3

Data presented in mean ± SD. **P < 0.001.

**Table 7:** The proportion of the various health levels and the average of the two participating doctor's VAS scores for each SC type (one-way ANOVA test).

Health state	Sasang constitution type		
	TE	SE	SY
Total (*n*)	100	72	126
Healthy (*n* (%))	28 (28%)	29 (40%)	58 (46%)
Unhealthy			
Subhealthy (*n* (%))	38 (38%)	24 (33%)	45 (36%)
Diseased (*n* (%))	34 (34%)	19 (27%)	23 (18%)
VAS score (mean ± SD)***	61.2 ± 23.2	67.6 ± 23.7	74.3 ± 19.9

***P value < 1*E* − 4.

**Table 8:** The significant variables and their coefficients (*α*) for the SHI_comp_ model of individual diagnostic components, as determined by the LASSO regression method.

SC type	Face		Pulse		Skin		Voice		Questionnaire	
	Variable	*α*	Variable	*α*	Variable	*α*	Variable	*α*	Variable	*α*
TE	(Intercept) FhL_Cr_std ChLD_Y_std Nose_Y_avg	63.28 2.75 −1.18 3.36	(Intercept) L_PVI L_PPW_PVI L_Adias_HR75 L_PSD_w3_w1 R_PDI R_Adias_HR75 R_PSD_w7_w1	59.89 1.62 −2.71 2.53 3.74 −2.19 0.56 0.54	(Intercept) Wrinkle_hand Wrinkle_arm_L *V*_*E*_	61.22 −4.98 −1.45 0.42	(Intercept) aPPQ eBW1 eF2 oF2 oBW2 sF10	61.23 −4.54 −4.29 0.29 1.08 −1.24 4.07	(Intercept) Exercise capability Digestion Sleep habits Extroversion and broad-mindedness	61.23 4.65 0.30 3.78 2.45

SE	(Intercept) FhL_Y_avg FhL_Y_std FhW_Y_std ChRU_Cb_std ChRW_Cb_std ChLU_Cr_std	68.53 0.89 −0.26 −7.35 −3.76 −0.16 −2.24	(Intercept) L_PDI	68.90 −4.83	(Intercept)	67.71	(Intercept)	67.60	(Intercept) Self-estimation of health state Exercise capability Digestion Sleep habits Unhealthy period	67.60 2.52 7.36 3.02 2.06 3.83

SY	(Intercept) FhR_Cb_std ChRU_Cb_avg ChRD_Y_std	74.35 −1.87 1.19 −3.07	(Intercept) L_t4 L_Aall_HR75	73.15 1.16 0.15	(Intercept) *E *	73.98 1.88	(Intercept) sFHL	74.28 −1.79	(Intercept) Self-estimation of health state Discomfort after eating, defecation, and urination Eating habits Extroversion and broad-mindedness Unhealthy period	74.28 4.11 1.46 −4.01 −2.49 0.92

**Table 9:** The final Sasang Health Index (SHI_sum_) based on the LASSO regression method, with the determination coefficient (*R*-square and adj. *R*-square) based on a 10-fold cross-validation test.

	TE		SE		SY	
	*β* (s.e)	*P* value	*β* (s.e)	*P* value	*β* (s.e)	*P* value
(Intercept)	−279.8 (75.7)	0.0029**	−187.6 (69.8)	0.0138*	−305.3 (192.3)	0.1181
Facial score	1.298 (0.668)	0.0587	0.886 (0.410)	0.0424*	1.710 (0.571)	0.0041**
Pulse score	0.784 (0.404)	0.0589	0.787 (0.698)	0.2720	0.193 (1.887)	0.9191
Skin score	0.580 (0.431)	0.1855	—	—	0.549 (1.368)	0.6895
Voice score	1.385 (0.285)	0.0000***	—	—	2.367 (1.175)	0.0487*
Questionnaire score	0.233 (0.383)	0.5463	0.874 (0.269)	0.0039**	1.609 (0.385)	0.0090**

*R*-square	0.58		0.65		0.38	
Adj. *R*-square	0.51		0.56		0.30	

Corrected by age and BMI, *P < 0.05, **P < 0.05, and ***P < 0.005.

**Table 10:** Changes of diagnostic variables associated with health degradation for each SC type.

SC type	Diagnostic component	Associated changes of diagnostic variables with health degradation
	Complexion	Increased brightness variance in the cheek (ChLD_Y_std) and decreased brightness in the nose (Nose_Y_avg)
	Pulse	Increased pulse depth (R_PDI) increased amplitude of the pulse wave within the pulse volume (L_PPW_PVI), decreased proportion of the area of the pulse wave during the diastolic period (L_Adias_HR75), and decreased PSD in a high harmonic frequency (L_PSD_w3_w1)
TE	Skin	Decreased wrinkle density (Wrinkle_hand)
	Voice	Increased frequency variation (aPPQ) increased bandwidths of the 1st and 2nd formants (eBW1, oBW2), and shift of the fundamental frequency at the 10th percentile into a lower frequency (sF10)
	Questionnaire	Decreased exercise capability, worsened sleep habits, and decreased tendency toward extroversion and broad-mindedness

	Complexion	Increased brightness variance in the forehead (FhW_Y_std) increased variances of blueness and redness in the cheek (ChRU_Cb_std, ChLU_Cr_std)
SE	Pulse	Increased pulse depth (L_PDI)
	Questionnaire	Low self-estimated health state, low exercise capability, indigestion, worsened sleep habits, and increased unhealthy period

SY	Complexion	Increased blueness variance in the forehead (FhR_Cb_std) and increased brightness variance in the cheek (ChRD_Y_std)
	Skin	Decreased elasticity (*E*)
	Voice	Increased ratio of the frequency intervals between a high to the median and the median to a low fundamental frequency (sFHL)
	Questionnaire	Low self-estimated health state, improved eating habits, and increased tendency toward extroversion and broad-mindedness
